# Arbuscular Mycorrhizal Fungi and Compost-Based Biostimulants Enhance Fitness, Physiological Responses, Yield, and Quality Traits of Drought-Stressed Tomato Plants

**DOI:** 10.3390/plants12091856

**Published:** 2023-04-30

**Authors:** Fatima Ezzahra Soussani, Abderrahim Boutasknit, Raja Ben-Laouane, Rachid Benkirane, Marouane Baslam, Abdelilah Meddich

**Affiliations:** 1 Center of Agrobiotechnology and Bioengineering, Research Unit Labelled CNRST (Centre AgroBiotech-URL-CNRST-05), “Physiology of Abiotic Stresses” Team, Cadi Ayyad University, Marrakesh 40000, Morocco; 2 Laboratory of Agro-Food, Biotechnologies and Valorization of Plant Bioresources (AGROBIOVAL), Department of Biology, Faculty of Science Semlalia, Cadi Ayyad University (UCA), Marrakesh 40000, Morocco; 3 Laboratory of Plant, Animal, and Agro-Industry Productions, Faculty of Science, University Ibn Toufail, Kenitra 14000, Morocco; 4 Department of Biology, Faculty of Science and Techniques, BP. 509, Boutalamine, Errachidia 52000, Morocco

**Keywords:** tomato, AMF, compost, drought stress, growth traits, nutrient quality, tolerance mechanisms

## Abstract

Climate change-driven water resource constraints cause tomatoes to suffer from drought. The use of biostimulants has emerged as an important approach to enhancing resilience to drought. However, the roles of biostimulants in the physicochemical characteristics of tomatoes in response to drought are poorly understood. In this study, we evaluated the ability of arbuscular mycorrhizal fungi (AMF) and compost (versus NPK application) to improve the agro-physiology, yield, and fruit quality of tomato plants and their tolerance to drought by comparing them with conventional chemical fertilizers (NPK). Under drought conditions, plant growth traits associated with yield and fruit bioactive compounds (carotenoids: 73%; lycopene: 53%; polyphenols: 310%; and flavonoids: 158%) were increased in the AMF-tomato treatment. Compost significantly enhanced sugars (ca. 60%) and protein contents (ca. 20%). Moreover, AMF protected the photosynthetic apparatus from drought-induced oxidative stress, improved photosynthetic efficiency, leaf water potential, and osmolytes, and reduced malondialdehyde (MDA) and hydrogen peroxide (H_2_O_2_) accumulation by increasing peroxidase (POX) (140%) and polyphenol oxidase (PPO) (340%) activities compared to their controls. Our findings revealed that NPK is an important nutrient-based fertilizer for plant growth and development. However, its efficiency as a fertilizer is quite low. In addition, we highlighted different mechanisms mediated by AMF and compost, inducing drought tolerance in tomato plants.

## 1. Introduction

Rapid population growth and significant climate change pose a threat to global food security [1]. Climate change-induced droughts have become one of the most common and severe abiotic stress factors that hinder plant development, resulting in a significant loss of agricultural yields in many arid and semiarid countries [2]. About one-third of the agricultural lands worldwide experience an inadequate water supply [3]. Climate change has altered rainfall patterns, causing tomato plants, the second-most produced vegetable worldwide in terms of tonnage, to suffer from drought during certain periods. Despite its constantly increasing production, estimated at 177 million tons per year with a total production area of ca. 5 million hectares [4], tomato production is concentrated in a few regions highly affected by climate change-driven drought waves, which is expected to decrease by 6% in 2050 compared to the baseline period of 1980–2009 [4]. The limited water supply hampers the transport and availability of soil nutrients, which affects plant morphology, physiological characteristics, photosynthesis, biomass, yield, and fruit production. To fulfill plants’ nutrient requirements, farmers apply a considerable amount of chemical fertilizer [5]. Current agricultural management practices rely heavily on the continuous use of conventional chemical fertilizers (1.9 × 10^11^ tons are used globally), which are industrially modified compounds that are generally water-soluble and have high accessible nutrient concentrations [6]. Although conventional agriculture, including the over-use of fertilizers, has increased crop production [7], it has also threatened the environment, deteriorated soil fertility, worsened groundwater quality, and negatively impacted food quality and human health [8]. The slow recovery efficiency of chemical fertilizers poses significant economic, environmental, and ecological losses [5]. Innovative management practices/strategies that enable plants to withstand abiotic stresses are urgently needed globally to improve crop production and reduce the reliance on chemical fertilizers [9,10]. Among these strategies, the application of biostimulants, such as arbuscular mycorrhizal fungi (AMF) and compost, has emerged as a promising approach in agricultural initiatives to increase crop tolerance to severe environmental conditions [11,12,13]. The use of microbes and compost is considered a crucial and effective approach to improving nutrient use efficiency under sub-optimal conditions. Biostimulants ensure the sustainability of agroecosystems under environmental stress by boosting the uptake of essential nutrients, improving plant–water status and photosynthesis, and leading to higher-quality agricultural products [14,15,16]. Regulating drought stress in plants using AMF and/or compost is a complex process that involves diverse metabolic pathways and metabolites [15,16,17,18,19,20]. These biostimulants can benefit plants in various ways, such as acting as biofertilizers (promoting NUE), bioprotectors (reducing the effects of abiotic stresses), and bioregulators (increasing plant fitness, growth, physio-biochemical and productivity traits). Using bio-organic compounds is a suitable alternative to improve the physicochemical properties of the soil [15,21,22,23,24,25,26], as well as crop growth and yield [27,28,29]. Applying compost contributes to improving the stability of soil structure and its physical properties through increasing organic matter [30,31], total porosity, hydraulic conductivity, aggregate formation, and water-holding capacity [32,33], leading to changes in the soil microbiome and ultimately improving plant tolerance to abiotic stresses [31,34].

In this study, biostimulants derived from AMF (*Rhizophagus irregularis*) and/or compost (olive pomace and olive mill wastewaters) were tested on tomato (*Solanum Lycopersicum* L.) plants under optimal and water deficit conditions. Considering the positive effects of biostimulants on plant fitness and yield, this study aimed to: (i) identify the agro-physiological, agronomic, and fruit quality responses of tomato to individual and combined use of biostimulants; (ii) estimate the improvement in drought tolerance in tomato by biostimulants; and (iii) find an alternative to minimize or replace the use of chemical fertilizers. We argue that the implementation of biostimulants as an environmentally friendly cultural practice will enhance physiological indices and boost tomato production and functionality under water limitation.

## 2. Results

### 2.1. Mycorrhizal Colonization

**Figure 1:**
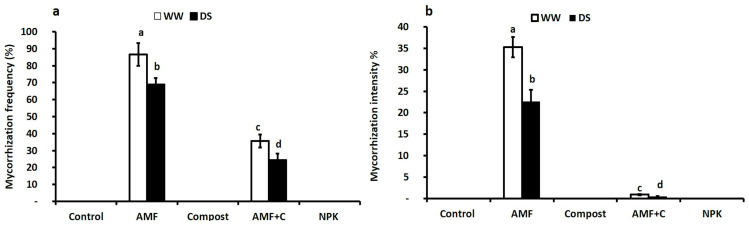
Effects of different water regimes (75% field capacity (WW); open bars and 35% FC; filled bars) on (**a**) mycorrhization frequency and (**b**) intensity in control plants (non-amended, non-inoculated), plants inoculated with arbuscular mycorrhizal fungi (AMF) and/or amended with compost (C), and plants treated with conventional chemical fertilizer (NPK). Data are mean ± SE of five biological replicates. Means that share the same letters are not significantly different at *p* < 0.05 (Tukey’s HSD).

The data indicate that no mycorrhizal structure was observed in the roots of the control, compost-treated, and NPK-treated tomato plants. Tomato plants inoculated with AMF alone under drought stress (DS) conditions showed a significant reduction in AMF infection frequency and intensity (F = 35.43; *p* < 0.001) and (F = 50.04; *p* < 0.001), respectively (see Supplementary Table S1 and Figure 1). Under DS, AMF-inoculated tomato plants exhibited higher root mycorrhization frequency and intensity (69% and 22%, respectively) compared to the dual treatment of AMF + C (25% and 1%, respectively). The interaction between AMF × C and AMF × Drought had a significant effect (*p* < 0.001) on mycorrhization intensity (see Supplementary Table S1).

### 2.2. Biostimulants Improved Tomato Growth Attributes and Reproductive Behavior under Normal and Water-Deficiency Conditions

Under well-watered (WW) conditions, application of AMF significantly improved shoot height (SH) by approximately 30% compared to non-inoculated and non-amended tomato plants. Drought stress resulted in marked reductions in shoot height, root length (RL), shoot dry matter (SDM), root dry matter (RDM), number of leaves (LN), number of flowers (NFL), number of fruits (NFr), fruit weight (FrW), and yield in comparison to WW conditions (Table 1). However, under drought stress, application of single or dual combinations of biostimulants (AMF and/or compost C) showed positive effects by promoting tomato growth traits to a greater extent than non-inoculated and non-amended treated plants (Table 1). Under DS, plants amended with compost alone showed the highest SH (14%), RL (75%), and LN (74%) values compared to controls. Moreover, the single application of AMF increased RL (83%) and NFL (67%) more than the controls. Under WW, plants treated with AMF + C significantly improved SDM by 13% (F = 34.63; *p* < 0.001) and RDM by 98% (F = 30.58; *p* < 0.001) in comparison with non-inoculated and non-amended tomato plants. NPK-treated plants showed the highest values of these parameters (ca. 25% in SDM and 290% in RDM) and the number of flowers (ca. 75%) in comparison with stressed control plants.

Similar to growth attributes, fruit weight and yield in tomatoes were significantly altered by water conditions and biostimulants application. Drought-stressed untreated tomato plants produced fewer numbers of flowers/fruits and smaller tomatoes than treated plants regardless of the soil water intensity. In the presence of mycorrhizal inoculation, fruit weight (F = 55.85; *p* < 0.001) and yield (F = 145.88; *p* < 0.001) were significantly improved, especially when plants were exposed to water shortage (Table S1). The application of AMF (F = 145.88; *p* < 0.001) and compost alone (F = 5.20; *p* < 0.05) significantly increased yield by 330% and 150%, respectively, compared to stressed control plants. The interaction between AMF × C × Drought had a significant effect (*p* < 0.001) on yield (Supplementary Table S1).

### 2.3. Biostimulants Enhanced Physiological Traits in Droughted Tomato

**Figure 2:**
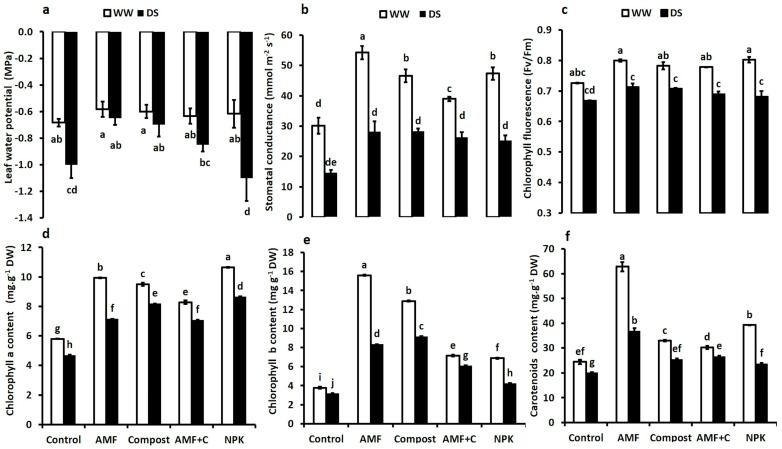
Effects of different water regimes (75% field capacity (FC) and 35% FC) and treatments (non-amended and non-inoculated control, biostimulants (AMF: arbuscular mycorrhizal fungi, C: compost, and AMF + C), and conventional chemical fertilizer NPK) on various physiological parameters of tomato leaves. Panel (**a**) Ψ_Leaf_: leaf water potential, (**b**) g_s_: stomatal conductance, and (**c**) F_v_/F_m_: chlorophyll fluorescence, (**d**) Chl a: chlorophyll a content (**e**) Chl b: chlorophyll b content, and (**f**) carotenoid content. Means sharing the same letters are not significantly different at *p* < 0.05, as determined by Tukey’s test.

All physiological parameters were significantly decreased by water limitation (*p* ≤ 0.05) (Figure 2). The leaf water potential (Ψ_Leaf_) values were negatively affected by DS compared to WW. However, the application of biostimulants significantly improved Ψ_Leaf_ of tomato plants under both water regimes. Under DS, plants treated with AMF had the least negative Ψ_Leaf_ values (+35%), followed by compost (+30%) and AMF + C (+15%) compared to non-inoculated and non-amended plants. Plants treated with NPK treatment showed the most negative values compared to the biostimulant-treated tomato plants (Figure 2a). Under water control conditions, there were obvious stomatal conductance differences between non-amended/non-inoculated and those treated with biostimulants or NPK. AMF-treated plants were the most effective in improving this parameter compared to control plants. Under water stress, g_s_ values decreased in tomato plants. However, the application of AMF and C + AMF significantly improved g_s_ in tomato plants by ca. 92 and 80%, respectively, compared to untreated control plants (Figure 2b). Chlorophyll fluorescence (F_v_/F_m_) was significantly decreased by drought. The application of biostimulants, especially AMF alone (+7%) significantly improved F_v_/F_m_ in tomato plants under DS conditions (F = 7.04; *p* < 0.05) (Figure 2c). The interaction between AMF × Drought had a significant effect for g_s_ (*p* < 0.01) and for F_v_/F_m_ (*p* < 0.05) (Supplementary Table S1).

Under water deficit, photosynthetic pigment content was significantly reduced (Figure 2d–f). However, the application of biostimulants and NPK fertilization increased pigment contents compared to control plants, regardless of applied water regimes. NPK fertilization was the most effective treatment to increase Chl a content under both WW and DS. The application of AMF and compost improved Chl b, especially under DS. Carotenoids were positively affected by AMF compared to the other treatments. The interaction between AMF × Drought, C × Drought, NPK × Drought, and AMF × C × Drought had a significant effect (*p* < 0.001) for Chl a, Chl b, and carotenoids (Supplementary Table S1).

### 2.4. Biostimulants Rescue Oxidative Stress Levels and Antioxidant Enzyme Activity in Droughted-Tomato

**Figure 3:**
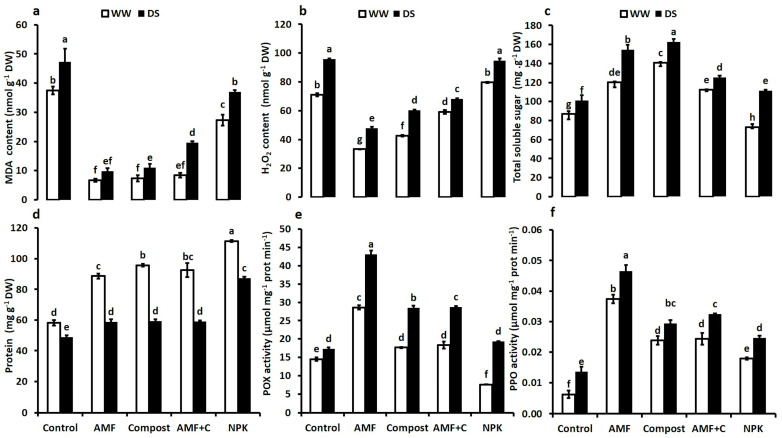
(**a**) MDA: malondialdehyde content, (**b**) H_2_O_2_: hydrogen peroxide content, (**c**) total soluble sugar content, (**d**) protein content, (**e**) POX: peroxidase activity, and (**f**) PPO: polyphenol oxidase activity in tomato plants leaves grown under two different water regimes (75% field capacity (FC) shown in open bars, and 35% FC shown in filled bars) and treated (or not; non-amended and non-inoculated control) with either biostimulants (AMF: arbuscular mycorrhizal fungi; C; compost; and combination of AMF + C) or conventional chemical fertilizer (NPK). Means sharing the same letters are not significantly different at *p* < 0.05, as determined by Tukey’s HSD.

To assess the damage caused by drought, we conducted analyses of malondialdehyde (MDA) and hydrogen peroxide (H_2_O_2_). Tomato plants exposed to water deficit stress showed an increase in MDA and H_2_O_2_ content (Figure 3a,b). Under DS, the application of biostimulants, alone or in combination, led to reduced MDA and H_2_O_2_ content compared to NPK-treated and non-inoculated/non-amended control plants. The single application of AMF or compost and their combination reduced MDA by ca. 80%, 77%, and 60% compared to the controls under DS. Similarly, the microbial and organic applications of biostimulants had a significant effect on H_2_O_2_ content (−50% in AMF, −37% in C, and −30% in AMF + C compared to the controls). The interaction AMF × C × Drought had a significant effect for MDA (F = 24.74; *p* < 0.001) and H_2_O_2_ (F = 88.93; *p* < 0.001) content (Supplementary Table S1).

Figure 3c–f present the data on the impact of drought and biostimulant applications on the total soluble sugar and protein content, as well as the activities of peroxidase (POX) and polyphenol oxidase (PPO) in tomato plants. Exposure to water deficit significantly increased the TSS and reduced protein contents in all the applied treatments. Under DS, biostimulants maintained a higher TSS content than non-amended and non-inoculated control plants. Indeed, the compost (+61%) and AMF alone (+50%), and bi-combination AMF + C (+25%) showed the highest values of this trait under water deficit, compared to NPK-treated (+10%) and non-inoculated and non-amended controls (Figure 3c). Under water-deficiency conditions, conventional fertilization showed the most significant increase (F = 40.60; *p* < 0.001) in protein content (+78%) in tomato leaves, followed by compost alone (+21%) or combined with AMF (+21%) (Figure 3d). The interaction AMF × C × Drought, NPK × Drought, and C × Drought had a significant effect (*p* < 0.001) for proteins content (Supplementary Table S1).

The DS led to an increase in peroxidase and polyphenol oxidase activities in tomato plants leaves compared to WW conditions. Consequently, compared to control plants, the application of biostimulants, particularly AMF alone, significantly enhanced POX activity by 150% after prolonged drought (Figure 3e). Similarly, biostimulants improved PPO enzyme activity under these conditions. PPO levels increased significantly in tomato plants inoculated with AMF alone (ca. 340%), AMF + C (140%), compost (115%), and NPK (80%) compared to non-mycorrhizal and compost-free treatments (Figure 3f).

### 2.5. Biostimulants Altered the Quality Parameters in Tomato Fruits

**Figure 4:**
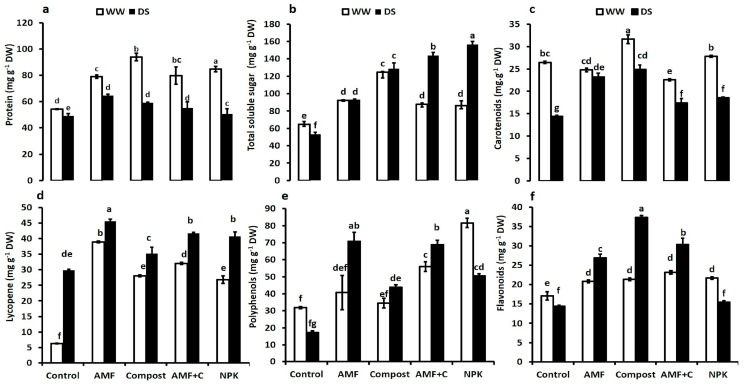
(**a**) Protein content, (**b**) total soluble sugar content; (**c**) carotenoids content, (**d**) lycopene content, (**e**) polyphenols content, and (**f**) flavonoids content in tomato fruit grown under two different water regimes (75% field capacity (FC) shown in open bars, and 35% FC shown in filled bars) and treated (or not; non-amended and non-inoculated control) with either biostimulants (AMF: arbuscular mycorrhizal fungi; C; compost; and combination of AMF + C) or conventional chemical fertilizer (NPK). Means sharing the same letters are not significantly different at *p* < 0.05, as determined by Tukey’s HSD.

The protein content, TSS, carotenoids, lycopene, polyphenols, and flavonoid content of tomato fruit juice were significantly affected by drought and biostimulant application (Figure 4a–f). The response of quality traits was similar for both treated and untreated plants. Drought stress reduced the protein content and increased the total soluble sugar content (TSS), except for controls, and antioxidant traits. Under normal water conditions, compost-treated plants had the highest protein level in fruits (ca. 73%). The protein content was significantly lower under drought stress, regardless of the (bio)fertilization application. However, under this DS, protein content in tomato fruit was higher in AMF-inoculated (31%), followed by compost-amended (21%), and bi-combination AMF + C (12%) treated plants compared to controls (Figure 4a).

Water exposure increased TSS in tomato fruit juice treated with biostimulants or NPK compared to untreated control plants (Figure 4b). Under this condition, NPK, AMF + C, compost, and AMF increased TSS by ca. 195, 170, 142, and 75%, respectively, compared to controls. The results for carotenoids (Figure 4c) and lycopene (Figure 4d) content in tomato fruits showed that nutrition variants and water levels altered the metabolism of these antioxidants. Under water deficit, carotenoid content significantly increased by compost alone (73%), AMF alone (62%), AMF + C (30%), and NPK nutrition (20%) compared to non-treated plants (Figure 4c). Similarly, under DS, biostimulants and NPK nutrition induced a statistically significant increase in lycopene content than compost-free and -AMF plants (Figure 4d).

Under water-control conditions, NPK led to a significant increase in polyphenols in tomato fruits (Figure 4e). Exposure to water deficit caused a significant decrease in this trait in untreated and NPK-treated plants compared to WW plants (Figure 4e). The same trend was observed in flavonoid content (Figure 4f). Biostimulants alone or combined increased polyphenols under DS compared to untreated or NPK nutrition. Under DS, AMF alone or combined with compost (AMF + C) yielded a significant increase in polyphenols content (ca. 310% and 295%, respectively) compared to stressed control plants (Figure 4e). The results showed that biostimulant application enhanced flavonoid content under water limitation. Compost-treated plants displayed the highest flavonoid values (ca. 160%), followed by AMF + C (110%), AMF (87%), and NPK nutrition (7%) compared to untreated stressed plants (Figure 4f). The interaction AMF × C × Drought and NPK × Drought had a significant effect (*p* < 0.001) for protein, TSS, carotenoids, lycopene, polyphenols, and flavonoid fruit content (Supplementary Table S1).

### 2.6. Heat Mapping for Growth, Physiological, and Biochemical Traits in Leaves and Fruit Quality of Tomato

**Figure 5:**
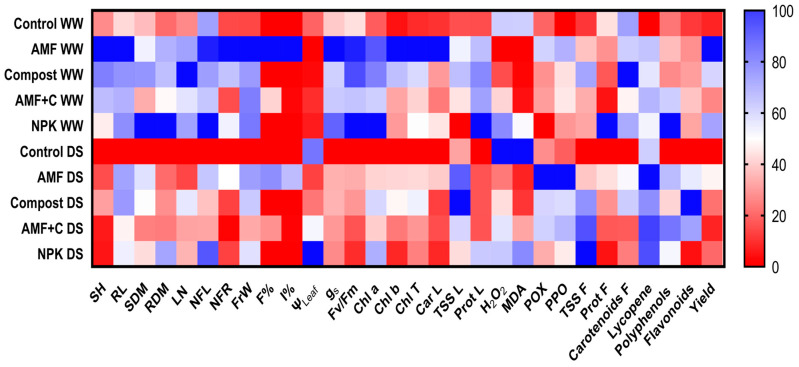
Heat map analyses of tomato plants (leaf and fruit) subjected to different treatments. SH: shoot height; RL: root length; SDM: shoot dry matter; RDM: root dry matter; LN: leaves number, NFL: number of flowers; NFr: number of fruit; FrW: fruit weight; F%: frequency of mycorrhization; I%: intensity of mycorrhization; Ψ_Leaf_: leaf water potential; g_s_: stomatal conductance; F_v_/F_m_: chlorophyll fluorescence; Chl a: chlorophyll a; Chl b: chlorophyll b; Car L: carotenoids in leaf; TSS L: total soluble sugar in leaf; Prot L: protein in leaf; H_2_O_2_: hydrogen peroxide in leaf; MDA: malondialdehyde in leaf; POX: peroxidase activity in leaf; PPO: polyphenol oxidase activity in leaf; TSS F: total soluble sugar in fruit; Prot F: protein in fruit; Carotenoids; Lycopene; polyphenols; flavonoids; and yield. In the heat map, the color gradient sets bright red as the minimum value, dark blue as the maximum value, and white as mid-range value, with a gradual transition (or gradient) between these endpoints.

The heatmap illustrates the relative performance of treated plants under WW and DS conditions. The darker blue color represents the highest values, while the darker red bands represent the lower values of tomato under both conditions (Figure 5). SH, RL, SDM, RDM, LN, NFL, NFR, FrW, and yield values showed moderate trends under DS conditions in plants treated with single AMF and compost versus untreated controls.

Water limitation resulted in higher values of AMF frequency and intensity in AMF-inoculated tomato compared to well-watered mycorrhizal plants. Tomato plants cultivated under normal or stress conditions exhibited significant differences in their physiological features, such as g_s_, F_v_/F_m_, Chl a, Chl b, and carotenoids. Among the various treatments tested, AMF treatment was found to be the most beneficial in enhancing these parameters under WW conditions compared to plants that were not inoculated or amended. Under DS, leaf water potential and stress markers (MDA and H_2_O_2_) were found to be higher in control plants and NPK. The single application of AMF was correlated with enzyme activities (PPO and POX), demonstrating their effective role in response to water deficit stress (Figure 5).

The quality of fruit (protein, carotenoids, lycopene, polyphenols, and flavonoids) was associated with AMF and compost alone or in combination, irrespective of water regime, with higher values observed under water shortage. The organic osmolytes (TSS) of leaves and fruit showed the highest intensities with increasing water deficit (Figure 5).

## 3. Discussion

The data revealed that drought stress significantly reduced root colonization, growth, and physiology/quality of tomato plants, as expected. Mycorrhization was less frequent and intense in tomato plants inoculated with AMF and amended with olive pomace compost (AMF + C) than in AMF alone. The increase in root colonization caused by AMF application alone can be explained by the soil’s low levels of organic matter and nutrients, particularly P. It has been reported that the introduction of vibrant AMF could help establish mycorrhiza earlier and maintain a higher inoculum potential than indigenous AMF populations, benefiting plant nutrition and development in the early growth stage [35]. AMF behavior is heavily influenced by environmental factors and soil management practices [36]. The colonization, which is better stimulated under limiting and poor soil conditions, was inhibited by P fertilization [37,38,39,40]. Compost inputs generally result in a decline in AMF colonization rate [13,18]. A double application of compost with a pure strain of *R. irregularis* caused a reduction in plant root colonization due to the large content of nutrients in the soil, causing the plant to become less dependent on AMF-acquiring (extra) nutrients [12,27,38]. The nature of the materials that go into the compost can influence the mycorrhization parameters. Here, the amount of OM and P in the compost may offset plant root colonization. Previous findings reported that olive mill wastewater margins-based compost negatively impacted the mycorrhizal frequency and decreased AMF hyphal length [41,42,43]. Nonetheless, compost-induced AMF root colonization reduction is not correlated with plant host growth stunt and physiology reduction.

Herein, the single and dual application of compost and/or *R. irregularis* significantly increased plant fitness, biomass, and the number and weight of fruits compared to untreated tomato plants. Root colonization by AMF has been extensively reported in improving plant growth and productivity under DS conditions. Inoculation of tomato plants with *Funneliformis mosseae* and *Rhizophagus irregularis* ensured higher DM than non-inoculated plants [44]. Extraradical hyphae growth has been linked to improved facilitation and transport of available nutrients and water at the root zone across the soil–plant continuum. Composting can improve nutrient availability and soil water retention, which benefits plant growth directly, especially when water is scarce. The combined use of AMF + C improved the plant’s above- and below-ground systems. Under water-stress conditions, the growth and yield performance of tomato plants treated with NPK, AMF, compost, and AMF + C may be due, at least in part, to mineral nutrition solubilization and absorption. The rate of nutrients, particularly P, influences photosynthesis, biosynthesis, and metabolite accumulation (i.e., proteins, sugars, phospholipids), as well as cell division, resulting in an increase in root and shoot biomass, leaf and flower number, and yield under water stress [45,46,47,48]. The use of conventional chemical fertilizers (NPK) may jeopardize fruit yield and tomato physiology under DS conditions by impeding the absorption and utilization of (other) nutrients When compared to biostimulants. AMF inoculation has been shown by Carillo et al. [49] to increase the number and weight of tomato fruits. The beneficial effects of AMF on tomato plants are related to mycorrhizal plants’ increased efficiency in absorbing soil nutrients [50,51,52,53]. According to Aini et al. [54], nutrients, particularly P and K, play an important role in increasing the quantity and quality of tomatoes.

The combinatorial benefit emancipated by the nutritional, physical, water status, and cellular effects of biostimulant-treated tomato plants resulted in a primary effect of producing more flowers and fruits and a greater degree of flower-to-fruit conversion process than untreated plants. The literature contains few reports of host plant flowering behavior changes caused by AMF colonization and compost application. Under DS conditions, flower abortion is a major constraint that leads to lower crop productivity, including tomato and maize (data herein, [46,55,56,57]). Under drought conditions, minerals and metabolites from the leaf (source) struggle to move to the developing fruits or kernels (sink), resulting in inhibition. However, the addition of biostimulants could have improved the nutritional and water status of the tomato plants, mitigating the negative impacts of drought and increasing fruit production, even under varying intensities of water stress.

Under these conditions, biostimulant treatments consistently increased fruit yields, and the water status levels of treated plants were much higher than the controls. Previous findings support biostimulant-aided host plant drought resistance and associated yield increase under DS [50,58,59,60]. Plants adapt to severe water stress by utilizing a mechanism that involve managing stomatal closure and opening, which ultimately reduces water loss [61]. As a result of a balance between water uptake and gas exchange, AMF-inoculated and/or compost-treated plants avoid dehydration as a consequence of DS [62]. Stomatal conductance (g_s_) decreased significantly in stressed tomato plants. Single and combined application of biostimulants contributed to a higher increase in g_s_ than uninoculated and unamended controls and NPK-treated plants. Tomato plants that have been inoculated with AMF and/or amended with compost exhibited an improvement of g_s_, which can be attributed to increased hydraulic conductance, enhanced root fungal uptake area, and improved osmotic adjustment [12,21,63,64,65]. The study’s finding indicated that higher mycorrhizal colonization and osmotic adjustment in plants treated with AMF and/or compost resulted in better plant water status under DS. This was evidenced by less negative Ψ_Leaf_ values compared to plants that received NPK treatment or remained untreated. AMF hyphae can enhance root hydraulic conductance under DS conditions by replacing the role of aquaporin activity [66]. In this line, Zou et al. [67] revealed that aquaporins in the roots of inoculated plants were up-regulated under DS, suggesting that AMF-colonized root systems actively maintain physiological water balance under DS [68,69]. Improvements in water status and g_s_ in compost-treated and mycorrhizal plants can lead to an increase in photosystem II (PSII) quantum yield (F_v_/F_m_) under severe DS conditions. The application of AMF and/or compost maintains plant efficient use of light energy at reaction centers in photochemical processes under water limitation [12,70]. Under DS, AMF-inoculated plants experienced an improvement in energy cycling between the reaction center and the chloroplast pool, an increase in the efficiency of excitation energy capture by PSII reaction centers, and a decrease in damage to PS reaction centers [70,71,72]. Droughted-tomato plants treated with NPK reduced water flux accompanying the soil–root–shoot pathway, leaf g_s_, F_v_/F_m_, and chlorophyll compared to biostimulant-treated plants. Compared to untreated and NPK-treated plants, the application of AMF and/or compost protected photosynthetic pigments and carotenoids from degradation under DS. Abbaspour et al. [73] suggested that untreated plants had less active physiology than AMF-inoculated plants under both WW and DS conditions, which is consistent with the findings mentioned above. AMF play a crucial role in mitigating the negative effects of DS by improving the chlorophyll and carotenoid content of plants [74,75,76,77]. Nutrient concentrations were higher in NPK treatment, which did not protect chlorophyll during DS exposure since this process requires a high leaf water potential (LWP). Under DS, LWP-induced stomata opening results in increased CO_2_ uptake and higher chlorophyll content [73,78].

Data revealed that DS led to an increase in sugar buildup in tomato fruits. Moreover, Ozbahce et al. [79] demonstrated that increasing water stress levels (50% FC and 25% FC) resulted in a proportional increase in TSS. This finding can be attributed to a decrease in fruit water accumulation without any significant change in the number of accumulated sugars [80,81]. Compared to WW, DS resulted in a significant decrease in carotenoids, sugar, and protein concentrations in tomato fruit. Regardless of the water regime, the application of biostimulants resulted in a substantial increase in these traits when compared to untreated plants. Similar to findings reported by Quiroga et al. [82] in maize, our data revealed that tomato fruit carotenoids were the most biofertilizer-induced trait under DS. The availability of nutrients may have a crucial role in the synergistic effect of AMF and compost in enhancing the total concentration of carotenoids [82]. Increased mineral release from the compost and improved mineral uptake by AMF may lead to high phytohormone levels and improved source-sink dynamics responsible for the rise in protein and sugar concentration. Previous studies have shown that beneficial microorganisms can enhance sugar metabolism- and protein system-related genes in plants under abiotic stresses [76,83]. According to Begum et al. [53], AMF inoculation of drought-stressed plants may have also increased the synthesis and decreased the breakdown of osmolytes, resulting in their significant accumulation. In a study by Ahanger et al. [84], crops with increased osmolyte accumulation showed superior growth performance and stress tolerance, as reflected in tissue water content and protein structure maintenance and functioning. The present study also demonstrates the biostimulant-mediated improvement in osmolyte accumulation, indicating the beneficial roles of AMF and compost in enhancing tomato fruit performance under water-deficient conditions. Osmolyte accumulation is thought to be a common response that accelerates water uptake under drought conditions. Under water-deficient conditions, the mycorrhizal and/or compost-amended tomato plants exhibited higher physiological metabolism than NPK-treated and control plants, leading to enhanced accumulation of organic osmolytes. This suggests that the application of biostimulants activates the natural physiological metabolism under DS. These findings align with previous studies indicating that tomato plants inoculated with AMF showed higher carbohydrate and protein content than controls under DS [85]. The accumulation of osmolytes in biofertilizer-treated plants under DS is attributed to the increase in net solute concentrations (or osmotic adjustment), which is a crucial mechanism for maintaining high plant water content and turgor. Tomato plants treated with biostimulants showed an increase in photosynthetic capacity, leading to improved organic osmolyte composition [86]. The accumulation of sugars in plant tissues could also be linked to the activation of starch hydrolysis during water stress, which is crucial in maintaining membrane integrity and facilitating the inflow of water to the cell [21,39,87,88]. Additionally, studies have indicated that AMF-treated plants under stress conditions exhibit up-regulation of sugar metabolism-related genes [53,89]. Similarly, the inoculation of tomato plants with AMF increased leaf protein levels under DS conditions, likely to maintain adequate hydration and turgor of the plant organs [13,90,91].

Under DS, plants inoculated with AMF and amended with compost showed an increased accumulation of soluble protein levels compared to untreated control plants. This increase in protein levels may explain the enhanced enzymatic antioxidant defense system observed in the former group [13,14,27]. By improving the antioxidant systems, plants are better protected against reactive oxygen species (ROS) that can damage cellular function by producing lipid-derived radicals and decreasing membrane stability [21,27,92,93]. The accumulation of ROS under droughts is due to chloroplast damage and the disruption of mitochondrial electron transport chains, which can lead to the degradation of membrane proteins by oxidative reactions or proteolytic activity in plants. Under DS, the increase in H_2_O_2_ content causes protein denaturation and lipid oxidation [59]. However, the application of AMF and/or compost under DS has been shown to reduce MDA and H_2_O_2_ production compared to untreated tomato plants, indicating the role of biostimulants in reducing oxidative damage in response to soil water limitation. Enhanced antioxidant enzyme levels play an important role in scavenging ROS and minimizing oxidative stress in plant cells under harsh conditions. Plants grown under DS and those inoculated with AMF or amended with compost showed a significant increase in POX and PPO levels compared to the controls.

The most important factor influencing consumer preference when purchasing tomatoes is their color, followed by size and juice content. Lycopene is a major bioactive component of tomatoes due to its health benefits [49]. Tomato color is affected by the proportion of lycopene and beta-carotene pigments during fruit ripening [94,95]. Despite the onset of drought stress, the antioxidant capacity of tomato fruits increased while lycopene levels remained stable. Atkinson et al. [96] reported that lycopene levels decreased under drought stress compared to well-watered conditions, while the amount of β-carotene increased. However, Carillo et al. [49] found that lycopene content increased by 27% in fruits grown under DS. Additionally, under DS conditions, AMF-inoculated plants had significantly higher lycopene levels (52%) compared to untreated plants. A recent study by Aguilera et al. [97] demonstrated that native AMF can improve tomato yield and lycopene concentration compared with controls. Furthermore, the addition of compost also increased lycopene levels in tomato fruit. Several studies have shown that using compost or AMF can improve the lycopene content of tomato fruits [98,99,100]. Wang et al. [101] reported that the increase in lycopene concentration was linked to the rise in potassium supply in tomato plants. Additionally, applying AMF and/or compost can enhance the assimilation of K^+^ in plants under stress conditions [12,102,103]. Agbede et al. [104] indicated that the increase in lycopene content in tomato fruits was attributed to the increased availability of nutrients, particularly K^+^ in the soil, due to organic matter mineralization. Moreover, K^+^ is essential for lycopene synthesis in tomato fruit since it influences the activity of the enzymes, pyruvate kinase and phosphofructokinase, and the formation of acetyl CoA, which is required for the production of isopentenyl diphosphate, the first precursor of carotenoids [105,106].

DS and biostimulants application progressively influenced polyphenols and flavonoids, which constitute 60% of total dietary phenolic compounds. The control plants showed a significant reduction in these parameters under DS compared to WW conditions. The single and combined application of AMF and compost significantly increased these metabolites more than stressed controls. AMF-inoculated tomato had higher polyphenols improvement (ca. 310%) in tomato fruit under DS. AMF inoculation is known to increase the levels of non-enzymatic antioxidants, which can help alleviate the impacts of water scarcity [107]. Both compost and AMF have shown significant enhancements in flavonoid content (ca. 160% and 87%, respectively). Mycorrhizal formation can modify the flavonoid profile by modifying the expression of phenylpropanoid-, flavonoid-, and isoflavonoid-related genes [59]. Flavonoids may also play a stimulating role in the growth of microorganisms such as AMF, thereby facilitating the fungus–host root encounters [108]. Strikingly, Fiasconaro et al. [109] revealed that peppers grown under drought had higher polyphenol levels in soil that had been composted. However, the role of compost in flavonoids and other phenolic acids is less clear. Some seminal works have observed considerable increases in phenolic compounds in the host due to AMF inoculation [110]. Plant–microbe interactions/symbiosis rely on these compounds that perform a range of functions. AMF-induced phenolic compound synthesis demonstrates the activation of the plant’s secondary metabolism. Phenolic compounds function as signaling molecules in the mutually beneficial relationship between plant and microbes, and they may boost the early stages of AMF formation. However, the host plant is expected to control root penetration and the development of AM through subsequent interactions with the fungal partner [110,111]. Under water-limited conditions, the drought resilience of mycorrhizal plants is attributed to an increase in phenolic metabolism as a response to mycorrhization. The plants respond more rapidly and generate significant amounts of phenolic chemicals that could affect the defense mechanisms. Furthermore, new phenolic molecules could be produced during the establishment of AMF colonization and function as signal molecules in the plant-AMF interaction [112]. Baliyan et al. [113] observed significant antioxidant activity (DPPH activity) for ROS scavenging through the accumulation of phenolic compounds under stressful conditions. The increased biosynthesis of plant phenols was observed in the plant during drought [114] and plays important physiological and biochemical roles in overcoming stresses, particularly those evoked by oxidative damage during drought stress [5,115].

## 4. Materials and Methods

### 4.1. Mycorrhizal Inoculum and Compost Preparation

The used AMF was a purified strain: *Rhizophagus irregularis* (isolate DAOM 197198) provided by the Plant Biotechnology Institute of Montreal (Canada). The propagules in the inoculum were enriched through co-cultivation with *Zea mays* L., which served as the host plant. The maize roots colonized by AMF containing hyphae, vesicles, and spores were cut into fragments of 1 cm and used to constitute the fungal inoculum. At the time of sowing, the tomato plants were inoculated by adding 30 g of inoculum (consisting of roots and substrate-containing spores) to their root system.

The number of AMF spores identified in this inoculum was 748 spores per 100 g dry soil. The non-mycorrhizal plants were given an equivalent amount of filtered inoculum to reintroduce other soil-free-living microorganisms along with the AMF, while the non-inoculated (and non-mycorrhizal) maize roots were added to maintain the ‘organic matter’ level in the pots. To obtain the filtrate, the mycorrhizal inoculum was passed through paper filters (Whatman, GE Healthcare, Buckinghamshire, UK) using 20 mL of distilled water.

The compost used in this study was produced from locally sourced olive pomace and olive mill wastewater, as previously described by Meddich et al. [116]. The compost had the following physicochemical properties: 9.3 pH, 9.98 mS cm^−1^ electrical conductivity (EC), 14.2% total organic carbon (TOC), 22.4% organic matter (OM), 652.08 mg/kg available P, and 8.51 g/kg total N. During transplantation, the compost was added to culture soil at a rate of 5% (*w*/*w*).

### 4.2. Biological Material, Application of (Bio)Fertilizers, and Experimental Design

Tomato (*Lycopersicon esculentum* Mill. cv. Campbell 33) seeds were sterilized in 10% sodium hypochlorite for 10 min, and then placed on 1% agar plates that contained 1/2 MS medium. The plates were incubated at 28 °C in darkness for 5 days. Seedlings were initially transplanted into plastic trays filled with sterilized peat. After 15 days, the seedlings with uniform growth were then transplanted into plastic pots containing either 5 kg of sterilized soil or 5% compost mixed with sterilized soil. The soil used for the experiment had a bulk density of 1.32 ± 0.01 g cm^–3^ and possessed the following characteristics: 8.2 pH, 0.14 mS cm^−1^ EC, 1% OM, 0.6% TOC, 8 mg/kg available P, 9 mg/kg total N, 2356 g/kg calcium, and 568 mg/g potassium.

The recommended doses of conventional chemical fertilizers (NPK) were applied at the following doses: 134 kg N/ha in the form of ammonium nitrate + 127 kg P_2_O_5_/ha as superphosphate + 332 kg K_2_O/ha as potassium sulfate [117].

The experiment involved five treatments to study the effects of biostimulants: (i) control without AMF inoculation or compost amendment; (ii) AMF alone: tomato plants inoculated with 30 g of AMF inoculum containing ca. 100 spores and 2 g of mycorrhizal root fragments consisting of hyphae, vesicles, and spores (AMF infection frequency = 96% and AMF infection intensity = 60%); (iii) C: compost alone (5% (*w*/*w*) with respect to culture soil); (iv) C + AMF: plants treated with both AMF and compost; and finally (iv) NPK: plants treated with conventional chemical fertilizer.

The tomato seedlings were maintained at 75% FC for a month before being subjected to two different watering regimes: 75% FC (WW; well-watered) and prolonged 35% FC (DS; severe drought stress) conditions. Soil moisture was measured twice a day using a TDR meter (Delta UK Ltd., Clacton-on Sea, UK). The volume of soil water needed under different growth conditions was calculated based on soil water content, soil bulk density, soil moisture maximum FC, and soil weight. The tomato seedlings were grown in a semi-controlled greenhouse with natural light (average photon flux density 410 µmol m^−2^ s^−1^), an average temperature of 25.5 °C, and an average relative humidity of 68.5%. The position of the pots in the greenhouse was rearranged every week to minimize the potential effects of any local or variable microclimatic conditions. The harvested fruits were stored at −20 °C until further analysis.

### 4.3. Growth and Mycorrhization Assessments

After 4 months, the effect of compost, AMF, and NPK on plant fitness was assessed by measuring the shoot height (SH), root length (RL), number of leaves (LN), number of flowers (NFL), number of fruit (NFr), fruit weight (FrW), and shoot (SDM) and root (RDM) dry matters. The tomato plants were pinched out young shoots and 1–2 trusses were left to develop for fruit harvesting.

Tomato root samples were washed, cleaned with 10% of KOH at 90 °C for 2 h, acidified with 5% HCl for 20 min, and stained with Trypan blue [118]. The microscopic estimation of AM fungal colonization was performed following the procedure of Trouvelot et al. [119]. The method described by Derkowska et al. [120] was employed to determine the frequency of mycorrhizal colonization (F%) and the intensity of mycorrhizal infection (I%) in the root system. For each sample, 15 randomly selected root fragments (each 1 cm in length) were used and the process was repeated five times per glass slide.

$$F\left(\%\right)=\frac{\left(Infectedrootsegments\right)}{totalrootsegments}\times 100$$

$$I(\%)=\frac{\left(95n5+70n4+30n3+5n2+n1\right)}{Totalrootsegments}$$

where n represents fragments with an index of 0, 1, 2, 3, 4, or 5 with the following infection rates: 100  >  n5  >  90; 90  >  n4  >  50; 50  >  n3  >  10; 10  >  n2  >  1; and 1  >  n1  >  0.

### 4.4. Physiological Traits Determinations

The mature, fully expanded leaves (five plants per treatment) located at the upper part of the stem were used to measure the leaf water potential (Ψ_w_) at predawn (6 a.m.–8 a.m.) using a pressure chamber (Model 600-EXP Super Pressure Chamber, PMS instrument, Albany, OR, USA). The Ψ_w_ of the cut leaves was also measured over the same days and immediately after the gas exchange measurements.

Stomatal conductance (g_s_) was measured on the abaxial side per plant (five measurements per treatment) between 10 a.m. and 11:30 a.m. using a portable steady-state diffusion porometer (Leaf Porometer LP1989, Decagon Device, Inc., Pullman, WA, USA). 

Chlorophyll fluorescence (F_v_/F_m_) was measured by a fluorometer (OPTI-SCIENCE, OS30p, Hudson, NY, USA). The upper side of the second fully developed leaf from the apex was dark-adapted by obscuring it for 20 min. The F_v_/F_m_ was measured on a leaf area of 12.5 mm^2^ by transmission at 650 nm. The fluorescence signal was recorded for one second with an acquisition speed of 10 μs [121]. The measured F_v_/F_m_ value corresponded to the quantum yields (F_v_/F_m_  =  (F_m_ − F_0_)/F_m_), where F_m_ is the maximum quantum yield of dark-adapted leaves and F_0_ is the initial quantum yield.

The concentration of photosynthetic pigments (chlorophylls a and b, total chlorophyll, and total carotenoid) was determined spectrophotometrically in 80% ice-cold acetone extracts as described by Arnon [122].

The photosynthetic pigment concentrations were calculated using formulae as follows:

$$Chla\left(\frac{mg}{g}DM\right)=\left[12.7\left(DO\;663\right)-2.69\left(DO\;645\right)\right]\;*\;\frac{V}{1000}\;*\;DM$$

$$Chlb\left(\frac{mg}{g}DM\right)=\left[22.9\left(DO\;645\right)-4.68\left(DO\;663\right)\right]\;*\;\frac{V}{1000}\;*\;DM$$

$$TotalChlorophyll\left(\frac{mg}{g}DM\right)=\left[20.2\left(DO\;645\right)+8.02\left(DO\;663\right)\right]\;*\;\frac{V}{1000}\;*\;DM$$

$$Carotenoids\left(\frac{mg}{g}DM\right)=\left[DO\;480+0.114\left(DO\;663\right)-0.638\left(DO\;645\right)\right]\;*\;\frac{V}{1000}\;*\;DM$$

where OD = optical density; V = final volume of the extract, and DM = Dry mater.

### 4.5. Biochemical Traits Quantification in Leaf

Total soluble sugars (TSS) were determined in frozen leaf powder (0.1 g) in 4 mL of ethanol (80% *v*:*v*) according to Dubois et al. [123]. Supernatant (0.2 mL) was mixed with 0.25 mL of phenol and 1.25 mL of sulfuric acid. Each sample was boiled for 15 min and cooled, and the absorbance at 485 nm was determined. 

The measurement of total soluble protein and antioxidant enzyme activities was conducted as follows: 0.25 g of frozen leaf powder subsamples were homogenized with 1 M phosphate buffer (pH 7) with 5% polyvinylpolypyrrolidone (PVPP). The homogenate was then centrifuged at 18,000× *g* for 15 min at 4 °C, and the resulting supernatant was utilized for antioxidant enzyme activity [124]. 

The total soluble protein concentration was determined by the method by Bradford [125], with bovine serum albumin serving as the standard.

The assay of peroxidase (POX) enzyme was conducted as per the method of Hori and Ayako [126]. The conversion of guaiacol to tetraguaiacol (ε = 26.6 mM^–1^⋅cm^–1^) was measured at 470 nm. The reaction mixture (3 mL) contained 1 M phosphate buffer (pH 7.0), 20 mM guaiacol, 40 mM H_2_O_2_, and 0.1 mL of the enzymatic extract. The absorbance change of 0.01 unit/min was considered as one unit of POX activity.

The activity of the polyphenol oxidase (PPO) enzyme was measured by using the method of Hori and Ayako [126] at 420 nm. The assay solution contained 20 mM catechol in 0.1 M phosphate buffer (pH 7), and 100 μL of the enzymatic extract. PPO activity was expressed in enzyme unit/mg protein. 

The malondialdehyde (MDA) content was determined by homogenizing 0.25 g of frozen powder subsamples in 10 mL of 0.1% trichloroacetic acid (*w*/*v*) and centrifuging at 18,000× *g* for 10 min according to Dhindsa et al. method [127]. The resulting supernatant was mixed with 2 mL of 20% TCA containing 0.5% thiobarbituric acid and the absorbance was recorded at 532 nm, with non-specific turbidity at 600 nm corrected for. The MDA content was calculated as [MDA] = 6.45 (A_532_ − A_600_) − 0.56 A_450_.

The amount of hydrogen peroxide (H_2_O_2_) was determined by the method by Velikova et al. [128]. Frozen leaf powder (0.25 g) was homogenized with 5 mL of 10% TCA (*w*/*v*) and centrifuged at 15,000× *g* for 15 min. The supernatant (0.5 mL) was mixed with 0.5 mL of potassium phosphate buffer (10 mM, pH 7) and 1 mL of iodic potassium (1 M). After incubation, the absorbance was read at 390 nm and plotted against a standard H_2_O_2_ curve.

### 4.6. Evaluation of Tomato Fruit Quality

#### 4.6.1. Preparation of Methanolic Extracts

Two grams of tomatoes were mixed with 50 mL of 80% (*v*/*v*) with shaking at 150 rpm for 2 h at room temperature, centrifuged at 5000 rpm for 15 min at 4 °C, and then filtered through filter paper (Buckner funnel and Whatman No. 1). The amphiphilic compound methanol (methanol extract) was collected for subsequent analyses as described by Dhawan and Gupta [129]. The measurement of total phenolic, flavonoid, and carotenoid content was carried out on quintuplicate extractions. All analyses were performed in triplicate per each extract.

#### 4.6.2. Sugar and Protein Content of Tomato Fruit

To estimate the sugar concentration, 0.1 g of fruit tissues was homogenized with 8 mL of 80% ethanol and then centrifuged for 10 min at 5000 rpm. The supernatant (0.2 mL) was mixed with 0.2 mL of 5% phenol and 1 mL of H_2_SO_4_, and the absorbance was read at 485 nm [130].

Soluble protein content in fruit was determined according to Bradford [125]. Methanolic extract (0.1 mL) was added to 5 mL of Bradford reagent. The reaction mixture was homogenized and kept at 30 °C for 30 min. The absorbance was read at 595 nm.

#### 4.6.3. Determination of Antioxidant Compounds

Total carotenoid content was determined by the method described by Poyrazoğlu et al. [131]. Tomato fruit (1 g) was homogenized in a solution of ethanol, acetone, and n-hexane (1:1:2 *v*:*v*:*v*). The extract was thoroughly agitated, incubated for 30 min, and the absorbance at 450 nm of the top layer of hexane was recorded. The calculation of carotenoid concentration (expressed in µg of β-carotene/g FW) was based on the molar absorption coefficient of 139 × 103 L mol^−1^ cm^−1^.

Lycopene extraction and determination were performed using the method of Roldán-Gutiérrez and Luque de Castro [132]. The absorbance was read at 472 nm using a spectrophotometer (ultraviolet-1800; Shimadzu, Kyoto, Japan). The concentrations of lycopene were calculated by using the extinction coefficient of ε: 3450.

Total phenols content was determined using the Folin–Ciocalteu method, as described by Magalhães et al. [133]. In brief, methanol extracts were mixed with 1 N Folin–Ciocalteu reagent (1:10), and after incubation, Na_2_CO_3_ (7.5%) was added to the extracts. The absorbance was then recorded at 765 nm using a multimode microplate reader Fluostar Omega (BMG Labtech, Chicago, IL, USA). The concentration of total phenolics was calculated using a standard curve of GAE and expressed as mg GAE/100 mg FW.

Total flavonoid content was determined according to the method described by Al-Farsi and Lee [134]. The methanol extracts (200 μL) were mixed with 60 μL of 5% NaNO_2_ and 60 μL of AlCl_3_ (10%). Then, 400 μL of 1 M NaOH was added to the reaction mixture, and the absorbance was read at 510 nm using quercetin as the standard. The results were expressed as mg quercetin equivalents QE per 100 mg FW.

#### 4.6.4. Statistical Analyses

The results were expressed as mean values ± SE (standard error) of five independent biological replicates. The significant differences among the tested factors (AMF; A, Compost; C, NPK, and drought; D) and their interactions with 95% confidence were calculated using Tukey’s honest significant difference test (*p*  ≤  0.05) through SPSS 23.0 software (multifactorial analysis of variance (MANOVA), IBM, Armonk, NY, USA). The measured traits of tomato plants under WW and DS conditions were categorized into a heatmap using GraphPad^®^ Prism v9.0 software.

## 5. Conclusions

Altogether, combining functional, physiological, and biochemical traits sheds light on new knowledge about the effect of single and dual applications of AMF and compost on tomato growth and resistance under droughts. Our findings indicate that the watering regime throughout the growth cycle has an effect on the growth and fitness of tomato plants, as well as on the impact of AMF and compost on their performance. Particularly, AM colonization and compost addition improved drought resistance of commercial tomato plants because of enhanced water status, photosynthetic machinery, osmoprotectants, antioxidant potential, and bioactive compounds (phenols and flavonoids) in conjunction with lower H_2_O_2_ and MDA contents. Maintaining leaf water status along with enhanced nutritional status may have facilitated the translocation of nutrients/assimilates to the sink and mitigated the negative effects of drought on fruit production in tomato plants. Additionally, the application of biostimulants resulted in improved tomato fruit quality.

These results have important implications for sustainable organic agriculture. They suggest that using microbial communities and organic amendments can make tomato crops more drought-tolerant and reduce the need for chemical fertilizers. To explore these effects further, we recommend conducting field experiments with AMF and compost and using advanced omics techniques to study the molecular basis of how AMF colonization and compost application reduce the negative effects of drought stress. 

## Figures and Tables

**Table 1:** Effects of different water regimes (WW: well-watered (75% of field capacity (FC), DS: drought stress (35% FC)) on growth and yield-related traits of tomato plants with different treatments: control plants (non-amended and non-inoculated), compost-amended plants, arbuscular mycorrhizal fungi (AMF)-inoculated plants, AMF + compost-treated plants, and conventional fertilizer (NPK)-treated plants. SH: soot height, RL: root length, SDM: shoot dry matter, RDM: root dry matter, LN: number of leaves, NFL: number of flowers, NFr: number of fruits, and FrW: fruit weight.

	SH (cm)		RL (cm)		SDM (g)		RDM (g)		LN		NFL		NFr		FrW (g)		Yield (g/Plant)	
	WW	DS	WW	DS	WW	DS	WW	DS	WW	DS	WW	DS	WW	DS	WW	DS	WW	DS
Control	85.3 ± 1.5 c–e	76.3 ± 1.1 e	28.0 ± 2.6 d	20.0 ± 2.0 b–d	6.1 ± 0.6 cd	5.0 ± 0.5 d	1.6 ± 0.2 ef	0.9 ± 0.1 f	10.2 ± 1.2 de	7.7 ± 0.6 e	8.7 ± 1.2 a–c	5.0 ± 1.0 d	1.7 ± 0.6 a–c	1.3 ± 0.5 b	26.3 ± 1.0 e	21.4 ± 0.9 ef	43.9 ± 1.7 g	28.5 ± 1.2 h
AMF	109.6 ± 1.5 a	81.3 ± 1.2 de	39.0 ± 1.0 a	35.3 ± 0.6 ab	7.9 ± 0.9 ab	6.7 ± 0.2 a–d	3.1 ± 0.5 bc	1.6 ± 0.4 ef	14.3 ± 1.0 a–c	9.0 ± 1.0 e	10.0 ± 1.0 a	8.3 ± 0.6 a–c	4.0 ± 1.0 a	2.6 ± 0.5 a–c	56.4 ± 1.0 a	46.0 ± 1.5 bc	225.9 ± 4.0 a	122.7 ± 0.7 d
Compost	106.0 ±1.0 ab	86.6 ± 0.6 c–e	35.6 ± 2.1 ab	33.6 ± 0.6 a–c	7.3 ± 0.7 abc	6.5 ± 0.6 a–d	3.0 ± 0.4 bc	1.8 ± 0.3 de	17.2 ± 2.6 a	15.6 ± 2.1 ab	9.0 ± 1.0 a–c	7.0 ± 1.0 cd	3.0 ± 1.0 ab	1.6 ± 0.5 a–c	48.1 ± 1.5 bc	42.9 ± 1.9 c	144.5 ± 4.7 c	71.6 ± 1.8 f
AMF + C	97.6 ± 1.5 b–d	78.3 ± 1.5 de	32.6 ± 1.2 a–d	29.0 ± 2.0 cd	6.0 ± 0.3 cd	5.6 ± 0.5 cd	2.5 ± 0.2 cd	1.7 ± 0.1 d–f	13.0 ± 1.0 b–d	10.7 ± 0.6 c–e	8.3 ± 0.6 b–d	6.7 ± 0.6 cd	1.7 ± 0.6 a–c	1.3 ± 0.5 b	47.9 ± 1.3 a–c	32.9 ± 1.8 ab	79.9 ± 2.2 e	43.9 ± 2.5 g
NPK	91.6 ± 1.5 a–c	78.0 ± 1.0 e	34.0 ± 2.0 a–c	30.1 ± 2.2 cd	8.0 ± 0.6 a	6.3 ± 0.3 b–d	4.2 ± 0.1 a	3.4 ± 0.4 ab	14.3 ± 1.2 a–c	11.0 ± 1.0 c–e	10.3 ± 0.6 a	9.7 ± 1.2 a–c	3.7 ± 0.6 a	1.6 ± 0.57 a–c	50.2 ± 0.3 ab	41.0 ± 1.5 c	184.1 ± 1.1 b	68.5 ± 2.5 f

Values sharing the same letter are not significantly different at *p* < 0.05, as determined by Tukey’s test.

## Data Availability

Not applicable.

## Supplementary Materials

The following supporting information can be downloaded at: https://www.mdpi.com/article/10.3390/plants12091856/s1. Table S1: Result of multivariate analysis of variance (MANOVA) for independent variables, including AMF (A), Compost (C), conventional chemical fertilizers (NPK), and the interaction among them.
