# Supplementary material for: Arbuscular Mycorrhizal Fungi and Compost-Based Biostimulants Enhance Fitness, Physiological Responses, Yield, and Quality Traits of Drought-Stressed Tomato Plants
Source: Plants (Basel). 2023 Apr 30;12(9):1856. doi: 10.3390/plants12091856 (PMC10180964; doi:10.3390/plants12091856)
Supplement: Supplementary file 1 [file plants-12-01856-s001.zip › plants-2358801-supplementary.pdf]

Supplementary Table 1: Result of multivariate analysis of variance (MANOVA) for independent variables, including AMF (A), Compost (C), conventional chemical fertilizers (NPK), and the interaction among them.

Parameters	AMF (A)		C		NPK		Drought (D)		A x C		A x D		C x D		NPK x D		A x C x D	
	F value	P value	F value	P value	F value	P value	F value	P value	F value	P value	F value	P value	F value	P value	F value	P value	F value	P value
Mycorrhization frequency	1965.55	***	386.76	***	0.00	ns	26.68	***	386.76	***	35.431	***	1.85	ns	0.00	ns	1.85	ns
Mycorrhization intensity	1028.17	***	938.11	***	0.00	ns	37.68	***	938.11	***	50.04	***	44.105	***	0.00	ns	44.10	***
Shoot height	3.59	ns	1.56	ns	5.73	*	98.02	***	31.40	***	1.77	ns	0.860	ns	3.32	ns	8.66	ns
Root length	4.79	ns	0.00	***	3.07	ns	45.91	***	58.28	***	3.40	ns	2.703	ns	47.45	***	6.95	ns
Shoot dry matter	2.23	ns	0.02	ns	23.19	***	23.53	***	34.63	***	0.06	ns	1.596	ns	1.28	ns	0.40	ns
Root dry matter	10.94	**	14.07	***	216.74	***	67.32	***	30.58	***	0..27	ns	0.208	ns	0.00	ns	6.95	*
Number of leaves	4.93	*	40.5	***	15.83	***	28.78	***	33.35	***	4.93	ns	1.075	ns	2.14	ns	1.77	ns
Number of flowers	5.00	*	3.20	ns	36.10	***	10.56	*	16.20	***	3.20	ns	5.000	*	8.10	**	0.80	ns
Number of fruits	3.12	ns	1.12	ns	9.00	**	8.94	ns	10.12	*	1.12	ns	0.125	ns	4.00	ns	0.12	ns
Fruit weight	122.75	***	47.62	***	279.82	***	60.97	***	302.72	***	55.85	***	11.338	**	21.48	***	0.14	ns
Yield fruit	1908.13	***	372.09	***	3664.74	***	6447.21	***	7672.28	***	145.88	***	5.202	*	1135.06	***	878.97	***
Leaf water potential	3.63	ns	0.90	ns	0.11	ns	54.296	***	20.51	***	0.90	ns	0.227	ns	2.84	ns	6.87	**
Stomatal conductance	40.21	***	4.60	*	147.19	***	524.69	***	213.83	***	8.23	**	2.179	ns	2.08	ns	9.33	**
Fv/Fm	2.81	ns	0.25	ns	10.68	**	333.58	***	17.23	***	7.04	*	2.292	ns	10.99	**	0.14	ns
Chlorophyll a	619.54	***	1025.77	***	5330.73	***	1880.19	***	2727.00	***	86.85	***	62.512	***	56.54	***	112.49	***
Chlorophyll b	4622.45	***	1327.34	***	2388.66	***	13488.34	***	45789.36	***	1093.15	***	651.604	***	592.42	***	6070.35	***
Carotenoids	1698.06	***	496.92	***	396.71	***	1669.34	***	1905.97	***	186.99	***	219.169	***	149.60	***	382.55	***
MDA	417.67	***	365.52	***	159.49	***	165.33	***	737.101	***	0.09	ns	0.435	ns	6.04	*	24.74	***
H_2O_2	1303.52	***	22.23	***	15.25	***	3940.56	***	7845.848	***	28.31	***	0.767	ns	4.39	*	88.92	***
Total soluble sugar	13.42	**	256.70	***	1.11	ns	480.30	***	867.554	***	2.76	ns	4.612	*	45.48	***	26.39	***
Protein content	192.53	***	168.89	***	1552.01	***	1505.33	***	113.911	***	7.10	*	146.369	***	40.59	***	95.55	***
PPO	594.03	***	20.36	***	158.37	***	186.23	***	661.057	***	0.11	ns	1.036	ns	0.132	ns	3.10	ns
POX	756.72	***	16.17	***	28.47	***	1573.19	***	1161.83	***	22.72	***	49.578	***	96.88	***	177.85	***
Protein fruit content	18.18	***	64.32	***	78.68	***	452.54	***	129.76	***	0.04	ns	59.868	***	62.58	***	14.10	***
Total soluble sugar fruit	65.24	***	1048.12	***	981.08	***	642.20	***	248.56	***	133.23	***	158.367	***	422.53	***	49.76	***
Carotenoids fruit content	78.41	***	53.26	***	55.79	***	629.31	***	507.23	***	134.43	***	2.850	ns	14.62	***	71.24	***
Lycopene fruit content	1248.15	***	95.10	***	152.87	***	1547.54	***	512.77	***	75.37	***	64.732	***	36.81	***	137.53	***
Polyphenols fruit content	285.44	***	43.22	***	331.35	***	0.06	ns	6.25	*	55.63	***	1.013	ns	13.20	**	41.44	***
Flavonoids fruit content	23.52	***	363.72	***	3.92	ns	8.68	ns	135.57	***	524.61	***	31.849	***	128.29	***	49.62	***

ns: non significant, *Significant at P < 0.05, **significant at P < 0.005, ***significant at P < 0.001
